# Ion Channels Prominent at Key U.S. and U.K. Conferences

**DOI:** 10.1089/bioe.2019.0009

**Published:** 2019-06-14

**Authors:** Sophie Rose, Marc Rogers

**Affiliations:** Metrion Biosciences Ltd., Cambridge, United Kingdom.

**Keywords:** ion channels, drug discovery, electrophysiology, conferences

## Abstract

A summary of the presentations featured at two key ion channel-focused events. This includes the Ion Channels in Drug Discovery XIX Satellite Meeting held at the 63rd Annual Meeting of the Biophysical Society (2019) and the Cambridge Ion Channel Symposium, cohosted by AstraZeneca and Metrion Biosciences.

Ion channels continue to rise in prominence in both academic and commercial research laboratories. Metrion Biosciences, alongside Sophion Bioscience, Nanion Technologies, Evotec, and SB Drug Discovery sponsored the “Ion Channels in Drug Discovery XIX” satellite meeting held on Friday, March 1, 2019, at the Baltimore Convention Centre, as part of the 63rd annual meeting of the Biophysical Society. The meeting was attended by ∼100 delegates from a variety of backgrounds, including academia, pharma, small to mid-sized biotech companies, and the service industry. After a brief welcome and opening remarks from Niels Fertig, CSO of Nanion Technologies who highlighted the merits of automated patch clamp (APC) and the growing number of publications centered around APC data, David Dalrymple of SB Drug Discovery introduced the keynote speaker. This was Professor William Colmers from the University of Alberta, who focused his presentation on the role of NeuroPeptide Y (NPY) and I_h_ HCN1-mediated currents play in stress modulation across different regions of the brain. NPY may underlie behavioral stress resilience and long-term structural changes in neurons. Work continues into the biological role of NPY with regard to energy balance, obesity, anxiety, and cachexia.

Dr. Stephen Hess then presented a three-part overview of Evotec's use of APC platforms to support ion channel drug discovery. Stephen first described a hit profiling study of voltage-dependent K_v_1.3 allosteric modulators, followed by a study of the on–off rates of Na_v_ inhibitors using a complex assay protocol they perfected. Stephen finished by highlighting the emerging power of CryoElectron Microscopy (Cryo EM) as a tool for structure-based ion channel studies and drug discovery. In <5 years, at least one structure from each of the seven TRP families has been determined, and in total ∼117 human ion channel structures have now been elucidated.

The next speaker was James Ellis from Nocion Therapeutics, a preclinical stage biotech company developing novel small molecules to selectively silence nociceptors involved in cough, itch, pruritus, inflammation, and pain responses. Jim spoke about the mechanism-of-action and liabilities of local anesthetics such as lidocaine and explained that an ideal Na_v_ channel blocker should maintain efficacy across indications, be administered topically to minimize systemic exposure and central nervous system (CNS) redistribution, and be cell impermeant and devoid of painful or irritating TRPA1/TRPV1 agonism. Nocion Therapeutics is developing a strategy originally described by its Harvard cofounders whereby charged Na_v_ antagonists can selectively enter overactive neurons through the large pores of ligand-gated pain receptors (TRPx, P2X, and ASIC) activated by exogenous agonists or endogenous ligands and inflammatory mediators.

Tianbo Li then presented an overview of Genentech's work on Na_v_1.7, a highly validated and well-characterized pain target. A key challenge for Genentech is devising new chemical matter with high potency and selectivity, and here he presented a case study on Pro-Toxin II (ProTx-II), a tarantula cysteine knot peptide toxin. Through a combination of alanine mutant scanning of a Na_v_1.7-Na_v_Ab chimera, charge alterations across the binding face of ProTx II, and determination of the cryoEM structure of ProTx-II bound to the channel chimera, it was shown that ProTx-II binds electrostatically to specific residues in the S3–S4 linker of voltage sensor domain II (VSD2). This information was used to create higher affinity analogues of ProTx-II and another spider venom, demonstrating the potential to aid future Na_v_ channel antagonist design.

Hongkang Zhang then spoke on behalf of Qwell Therapeutics, a spinout from Q-State Biosciences, who works on nonopioid treatments for pain. Hongkang outlined the rationale for a new type of ion channel screening platform, comparing the costs, temporal resolution, mechanistic detail, and throughput available from conventional plate-based readers (e.g., fluorometric imaging plate reader) and APC machines. Qwell Tx is developing the single-site Optopatch optogenetic platform from Q-State into a higher throughput 24-well device suitable for target-based screening of ion channels. Using Na_v_1.7 channels, they have preliminary data to support claims for fast, sensitive, linear readouts of ion channel activity that combine the advantages of plate-based readers and APC platforms.

GABA_A_ receptors are a complex ligand-gated ion channel family with at least 16 subunits. They are important drug targets for anxiety and epilepsy, and the next speaker, David Dalrymple, highlighted SB Drug Discovery's efforts to create a suite of GABA_A_ receptor cell lines and validated APC screening assays. SB generated 19 human GABA_A_ receptor cell lines of various alpha 1–6, beta 1–3, gamma, and delta subunit combinations that they validated pharmacologically on the Syncropatch384 using stacked tip protocols. Several positive allosteric modulators were found after a plate-based screen of SB's compound libraries, most of which were confirmed by APC electrophysiology.

Professor Francesco “Pancho” Bezanilla was the afternoon keynote speaker, providing an overview of his recent work on genetically encoded voltage indicators and optocapacitance techniques to probe the structure and function of voltage-dependent ion channels and transporters. The study of gating currents requires new types of fast voltage indicators, and Pancho described his work with ASAP-1, an ADP-ribosylation factor GTPase-activating protein from chicken. Pancho then explained optocapacitance, whereby infrared light can depolarize biological membranes and excite muscle cells, *Xenopus* oocytes, and neurons. This local heating and activation can be focused spatially and in terms of tissue-penetrating wavelengths through application of gold nanoparticles, graphite, and carbon nanotubes to cells, and even directed to subcellular sites and specific receptors and channels through conjugation to toxins (e.g., Ts1 toxin for Na_v_) and antibodies (P2X and TRPx), enabling exquisite optical control of cellular excitability.

Marc Rogers, CSO of Metrion Biosciences, then presented an overview of an 8-year drug discovery collaboration with a global pharma partner, for which Metrion provided *in vitro* and *ex vivo* screening services using their manual patch and APC expertise. During the collaboration, they developed high-quality voltage-gated ion channel assays to reliably identify and profile novel, potent, safe, and efficacious state-dependent modulators of a pain-related ion channel target. This yielded a development lead compound and a back-up series with therapeutic potential equal or superior to current clinical treatments.

Iontas specializes in mammalian phage display and antibody discovery and affinity maturation. Aneesh Karatt-Vellatt summarized his work on Knottin-antibody fusion proteins (KnotBodies), a novel antibody format that enables the targeting of “difficult” drug discovery proteins such as ion channels and G-protein coupled receptors (GPCRs) by combining antibody light chains with inhibitor cystine knot toxins and other small peptides to achieve increased specificity and half-life. Iontas is using Na_v_1.7, ASIC1a, and K_v_1.3 channels as case studies, and Aneesh gave an overview of K_v_1.3 as a target for autoimmune disease, which affects 2–3% of the worlds' population and may have a market value ∼$45.5 Bn by 2022. As the sea anemone toxin analogue ShK-186 recently showed efficacy in a clinical trial on psoriasis, Iontas incorporated several K_v_1.3-targeting toxins into the knot body template, yielding potent and selective K_v_1.3 blockers capable of reducing T cell cytokine release.

Sam Goodchild then presented an overview of Xenon Pharmaceuticals' program to develop Na_v_1.6 modulators to treat epilepsy and rare forms of encephalopathy such as IEEE13. The Na_v_ inhibitors currently used have a low therapeutic index and are poorly selective, often being dosed at concentrations that can cause adverse side effects. Based upon their extensive work on aryl sulfonamide gating modifiers of Na_v_1.7 (in collaboration with Genentech), Sam described his precision medicine approach that identified two novel compounds that selectively target Na_v_1.6 (XPC-224) and Na_v_1.6/1.2 (XPC-462) through a greater than 1000-fold preference for the inactivated state *via* binding to specific residues in VSD-IV. The compounds are efficacious in mouse seizure models at 3 × *in vitro* IC_50_ and exhibit 100-fold TI over acute toxicity effects. Thus, these compounds promise to provide superior efficacy and side effect profile to current anticonvulsant medications such as phenytoin and carbamazepine.

The final speaker was Fern Toh from Alkermes, a global pharmaceutical company working primarily on CNS disorders including Multiple Sclerosis, schizophrenia, depression, and addiction. Alkermes is interested in developing higher throughput mechanistic and translation assays for CNS drug discovery. Fern outlined ongoing work to correlate molecular profiling and plate-based multielectrode array data from cultured rodent cortical and hypothalamic neurons with functional recordings of ion channels and receptors on an APC platform. Alkermes hopes to build upon these studies to look at GPCR modulators and human stem cell-derived neurons to aid the translation of screening results to CNS drug candidates.

AstraZeneca and Metrion Biosciences again joined forces in on April 9, 2019 to cohost the 6th Cambridge Ion Channel Forum, held at the Milstein building at Granta Park. The event was a key feature of the ion channel enthusiasts' calendar since 2011 and the 2019 meeting attended by ∼65 delegates who also enjoyed a networking lunch and poster session before the keynote talk presented by Professor Sarah Lummis from the University of Cambridge.

Sarah's group studied the molecular function of neurotransmitter-gated ion channels, with an emphasis on Cys-loop receptors. Her research is focused on both vertebrate and bacterial Cys-loop receptors, which includes nicotinic acetylcholine, gamma-aminobutyric acid (GABA), glycine, 5-HT_3_, and glutamate-activated Cl-receptors. Sarah's talk focused on the prokaryotic ELIC channel from *Erwinia chrysanthemi*, which is activated by GABA and cysteamine and triggers a stress signal or defense mechanism in plants. CryoEM data for ELIC can be used to probe the structure and function of GABA_A_ receptors. Sarah concluded with animal and human data on mutations in glycine receptors that cause startle disease or hyperkeplexia. These disturb inhibitory glycine-mediated neurotransmission but remain poorly understood.

The next speaker was Professor Martin Gosling, the CSO of Enterprise Therapeutics and Professor of Molecular Pharmacology at the University of Sussex. Enterprise Therapeutics focuses on the development of novel therapies for respiratory diseases by targeting the underlying mechanisms of mucus congestion, with a focus on Transmembrane Member 16A (TMEM16A) calcium-activated chloride channel and epithelial sodium channels. Martin presented a case study of TMEM16A, which has recently been proven to behave as a key orchestrator of anion secretion in human airway epithelium. The team started a parallel screening campaign to identify low-molecular weight potentiators of TMEM16A using plate-based readers and APC platforms, identifying EXT001 as being particularly efficacious. This TMEM16A potentiator significantly enhanced the secretory current in cystic fibrosis patient-derived human bronchial epithelial (HBE) cells, with EXT001 promoting mucosal fluid secretion to clinically relevant levels. In collaboration with both the Cystic Fibrosis Trust and Cystic Fibrosis Foundation, Enterprise are taking several TMEM16A potentiators into clinical development.

Paul Miller from the University of Cambridge then provided insights into the modulation of GABA_A_ receptors, a ligand-gated chloride channel that mediates fast inhibitory synaptic transmission in brain. As well as being a validated target for anxiety, sleep, and addiction, Sage Therapeutics received approval for its GABA_A_ modulator brexanolone in postpartum depression. GABA receptors possess well-known binding sites in the pore and near the ligand-binding pocket where allosteric modulators like diazepam can bind. There remains a challenge, however, to develop more selective ligands with diverse chemistry and mechanisms-of-action, and Paul described how his group, in collaboration with Professor Jan Steyaert, has recently raised llama nanobodies against the α1β3 GABA_A_ receptor. Binding of nanobodies to purified GABA_A_ proteins is detected using biacore/SPR and patch clamp electrophysiology, with one promising nanobody (Nb38) found to favor the GABA-bound activated state, acting as both an agonist and a potentiator. Binding of Nb38 helped elucidate the GABA_A_ receptor structure to 5 Å resolution, which was improved to 2.5 Å using CryoEM, revealing the two binding sites of Nb38 between the alpha and beta subunit interfaces. Paul explained that this study is being extended through generation of a 10^8^ nanobody library.

The session was brought to a close by Verity Talbot with an overview of AstraZeneca's efforts to develop new cardiac ion channel assays to meet the requirements of the Food and Drug Administration (FDA)'s CiPA initiative (Comprehensive *In Vitro* Proarrhythmia Assay). CiPA aims to promote a novel paradigm for assessment of clinical cardiac risk, especially Torsades de Pointes, by combining *in vitro* ion channel assays and *in silico* modeling techniques together with translational human tissue assays to facilitate early drug development and compound selection. Verity described how the FDA selected a training set of 28 compounds of known TdP risk that were screened by pharma companies, CROs (including Metrion), platform providers, academic collaborators, and regulatory agencies as part of the CiPA consortium. AstraZeneca selected 12 compounds from the toolbox and used them to validate an APC “dynamic hERG” assay utilizing the Milnes voltage protocol. They were able to generate kinetic parameters for use in *in silico* models of action potential duration and to determine qNet scores, finding that combinations of very different hERG binding parameters could estimate similar qNet scores of cardiac arrhythmia risk. Verity explained that these results are driving ongoing work to assess whether APC kinetic hERG data are predictive of proarrythmic risk and how AstraZeneca will use the CiPA paradigm in its drug discovery process.

Ion channels and bioelectricity are intertwined, and it was exciting and highly rewarding to hear some of the latest developments in the field and to see people from academia and industry come together and share their knowledge. We very much look forward to future meetings and to continuing these insightful discussions.

